# Supplementary material for: GolgiFold: A Structural Predictome of Human Golgi Protein Complexes
Source: bioRxiv. 2026 Oct 30:2026.10.29.758816. Preprint. [Version 1] doi: 10.64898/2026.10.29.758816 (PMC13734120; doi:10.64898/2026.10.29.758816)
Supplement: Supplementary file 1 [file NIHPP2026.10.29.758816v1-supplement-13.pdf]

bioRxiv preprint doi: https://doi.org/10.64898/2026.10.29.758816; this version posted October 30, 2026. The copyright holder for this preprint (which was not certified by peer review) is the author/funder, who has granted bioRxiv a license to display the preprint in perpetuity. It is made available under a CC-BY 4.0 International license.

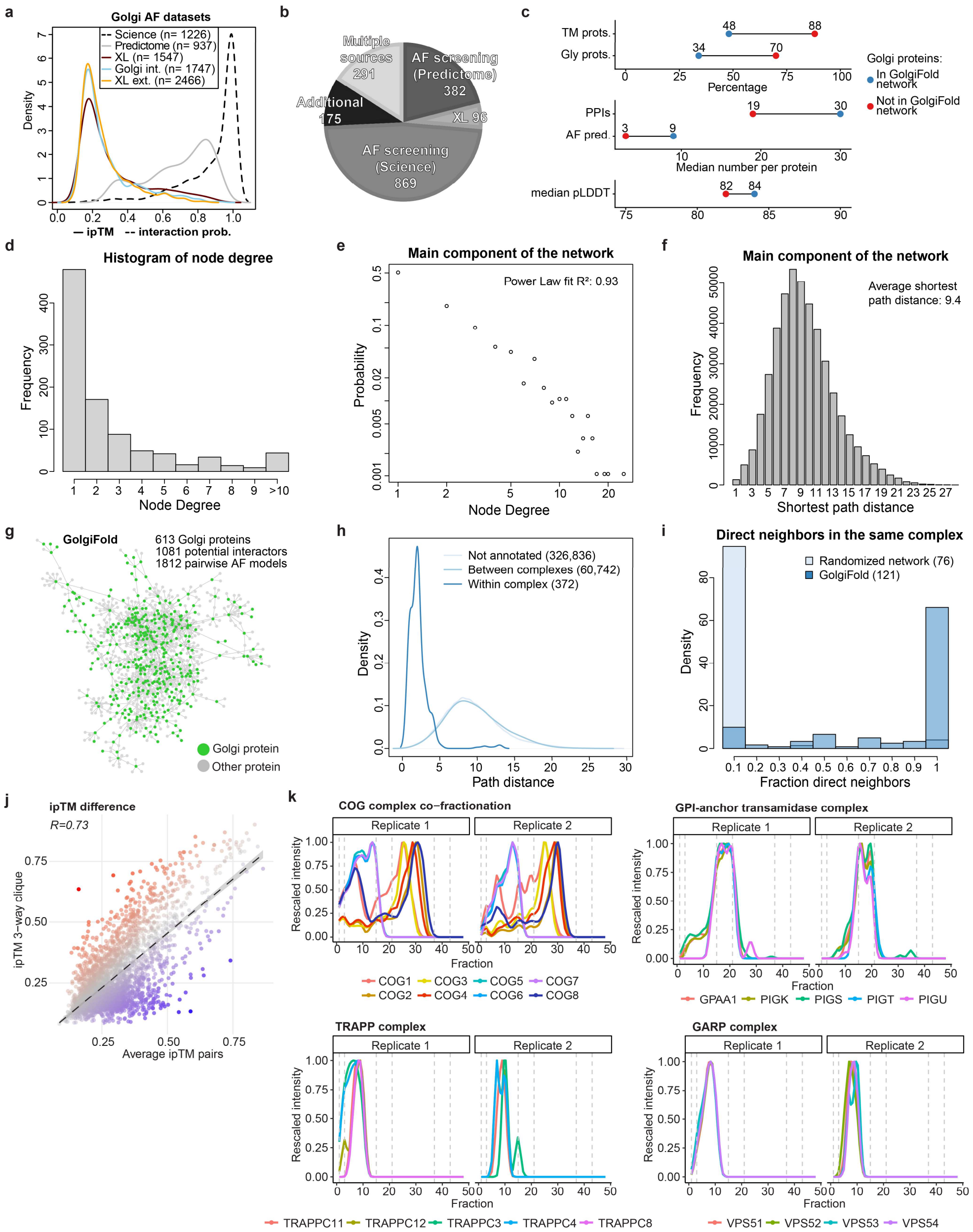

Supplementary Figure 2. Large-scale AF, network analysis and correlation profiling. a, Distribution of ipTM scores from AF predictions of Golgi interactions from each dataset. Interaction probabilities are indicated for Science AF screening since ipTM was not reported. **b**, Percentage of AF predictions included in GolgiFold from each dataset and source study. **c**, Comparison between Golgi-annotated proteins present (613) and absent (244) in GolgiFold. **d**, Degree distribution (number of edges per node) of GolgiFold network. **e**, Power law log-log plot showing the degree of a node (number of edges) and the probability of GolgiFold network. **f**, Distribution of the shortest path distances between all proteins in GolgiFold network. **g,** Core component (i.e. largest module) of the GolgiFold network containing 1,812 AF protein pairs. Green and grey nodes represent 529 Golgi and 1,081 other proteins, respectively. **h**, Distribution of path distances between proteins within and between the same complex compared with proteins without complex annotation. **i**, Distribution of fraction of direct neighbors in the same complex for each protein compared with a randomized network control. **j**, ipTM scores for AF predictions of all cross-linked 3-way cliques

bioRxiv preprint doi: https://doi.org/10.64898/2026.10.29.758816; this version posted October 30, 2026. The copyright holder for this preprint (which was not certified by peer review) is the author/funder, who has granted bioRxiv a license to display the preprint in perpetuity. It is made available under a CC-BY 4.0 International license.

compared to the average pairwise ipTM score of its components. Color gradient represents the score difference. **k**, Co-fractionation profiles of selected protein complexes from BN-MS.

bioRxiv preprint doi: https://doi.org/10.64898/2026.10.29.758816; this version posted October 30, 2026. The copyright holder for this preprint (which was not certified by peer review) is the author/funder, who has granted bioRxiv a license to display the preprint in perpetuity. It is made available under a CC-BY 4.0 International license.

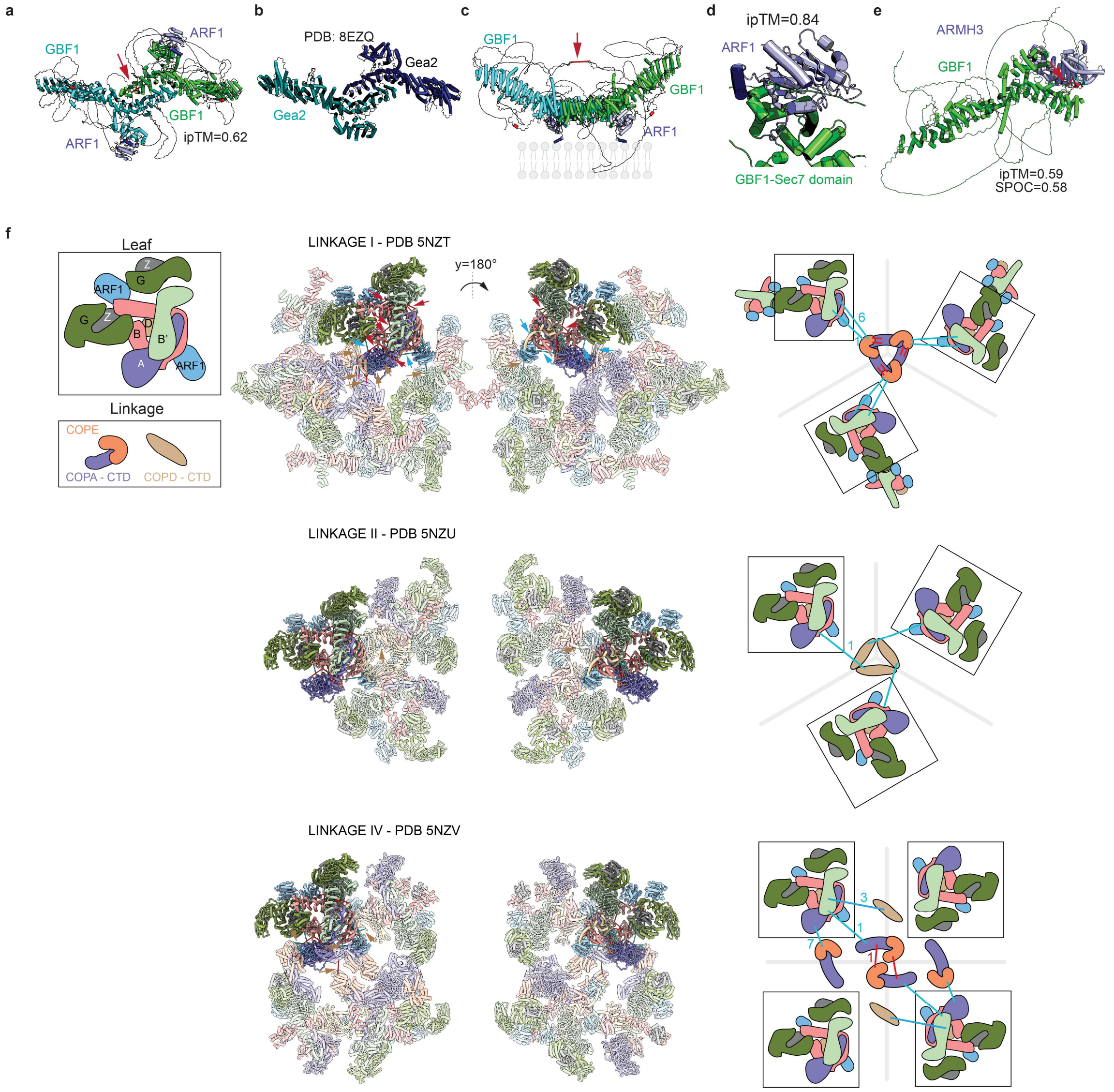

Supplementary Figure 3. Structural predictions and crosslink analysis of COPI coatamer complexes and ARF1 GEF. **a**, AF-M prediction for GBF1 dimer with two ARF1 molecules associated with the Sec7 GEF domain on each GBF1 monomer. Red arrow indicated the location of crosslink identified within the homodimer (**Supplementary Figure 1h**). ipTM (black font) and SPOC (gray font) scores are indicated for each pairwise prediction. **b**, Structure of yeast Gea1 homodimer (ortholog of GBF1, PDB: 8EZQ)[52]. **c**, $GBF1_2$-$ARF1_2$ prediction indicating the orientation of ARF1 N-terminal helices within the membrane. Red helices in GBF1 (residues 1079-1085) correspond to sequences identified in yeast that facilitate membrane interaction through amphipathic helix interactions[52]. Red arrow indicated the location of crosslink identified within the homodimer. **d**, Interface for predicted interaction of the GBF1 Sec7 GEF domain with ARF1. **e**, Pairwise AF-M prediction for GBF1 and ARMH3, with the indicated crosslink (red arrow) within distance constraint. Red β-turn shows residues 377-388 in GBF1 and the cyan β-strand shows residues 631-637 in ARMH3. **f**, Summary of COPI crosslinks across three linkage interfaces. The "leaf" schematic (top left) represents the core COPI assembly composed of A, B, B2 (B'), G_2, Z_2, and $ARF1_2$ subunits. Core COPI together with crosslinks are indicated along with two additional semitransparent leafs. Linkage I (PDB:5NZT)[54] represents a 3-fold symmetric organization around three $COPA^{CTD}$-COPE subunits. Linkage II (PDB:5NZU)[54] represents a 3-fold symmetric organization around three COPD subunits. Linkage IV

bioRxiv preprint doi: https://doi.org/10.64898/2026.10.29.758816; this version posted October 30, 2026. The copyright holder for this preprint (which was not certified by peer review) is the author/funder, who has granted bioRxiv a license to display the preprint in perpetuity. It is made available under a CC-BY 4.0 International license.

(PDB:5NZV)[54] represents a 4-fold symmetric organization around for $COPA^{CTD}$-COPE subunits. Red arrow heads point to a subset of crosslinks within distance constraints. Cyan arrows denote crosslinks beyond distance constraints. Brown arrows indicate crosslinks within linkage components not present in the core leaf.

bioRxiv preprint doi: https://doi.org/10.64898/2026.10.29.758816; this version posted October 30, 2026. The copyright holder for this preprint (which was not certified by peer review) is the author/funder, who has granted bioRxiv a license to display the preprint in perpetuity. It is made available under a CC-BY 4.0 International license.

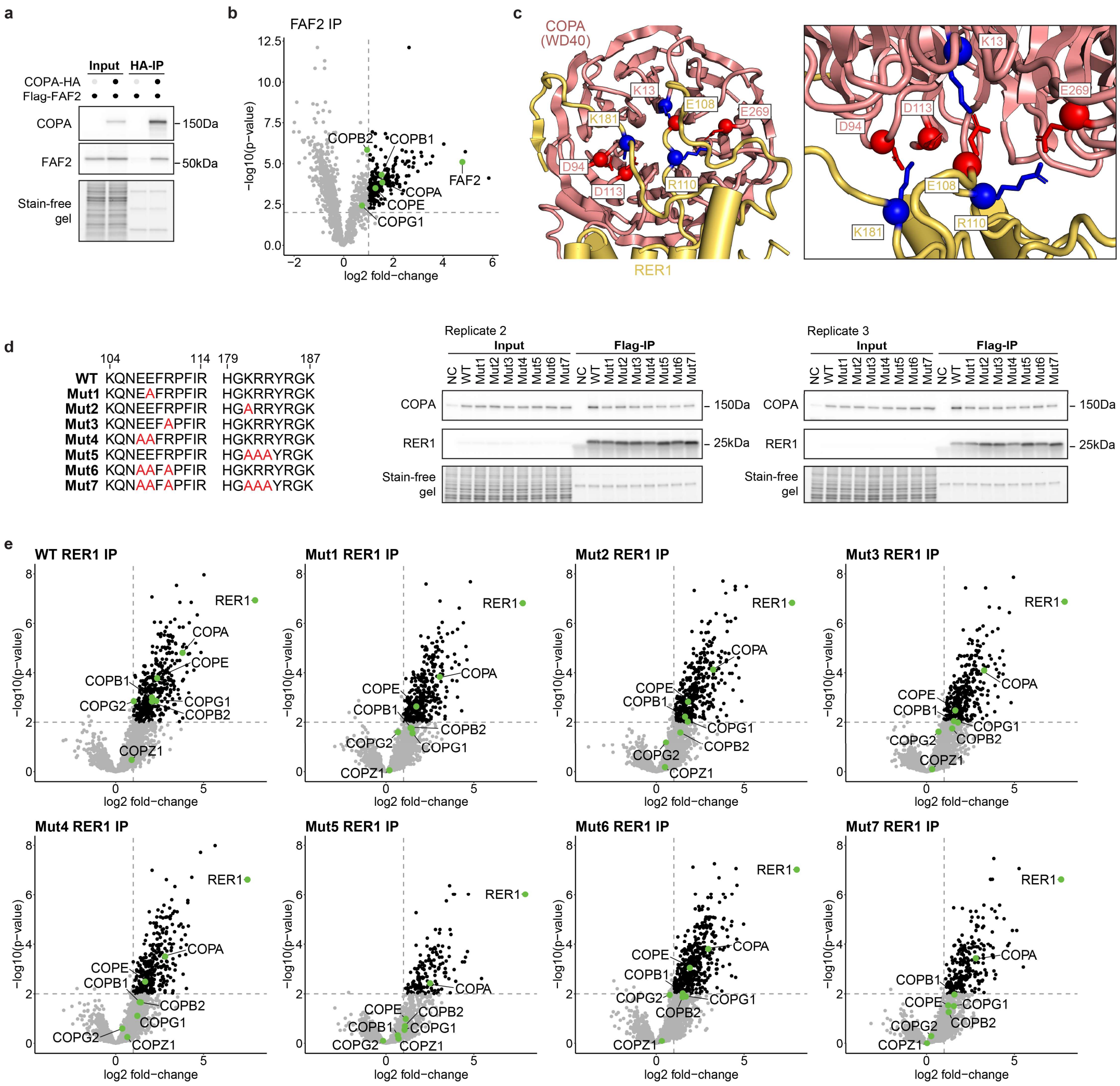

Supplementary Figure 4. COPI-RER1 interaction analysis. a, Flag-FAF2 co-precipitate with COPA-HA in transfected HEK293 cells, as examined using immunoblotting of anti-HA IP. **b**, Volcano plot showing the proteomic analysis of Flag-FAF2 IPs from transfected HEK293 cells. **c**, AF predictions for RER1 and COPA-WD40 domain. COPA-WD40 binding pocket and RER1 residues predicted to participate in the binding interface. Red and blue represent basic and acidic residues, respectively. **d**, Co-precipitation of COPA-HA with Flag-RER1 containing either WT or mutant sequence, as examined by immunoblotting of anti-Flag IP (three biologically independent replicates, together with **Figure 3h,i**). **e**, Volcano plot showing the proteomic analysis of IPs for WT and variant RER1 as quantified by mass spectrometry (related to **Figure 3j**).

bioRxiv preprint doi: https://doi.org/10.64898/2026.10.29.758816; this version posted October 30, 2026. The copyright holder for this preprint (which was not certified by peer review) is the author/funder, who has granted bioRxiv a license to display the preprint in perpetuity. It is made available under a CC-BY 4.0 International license.

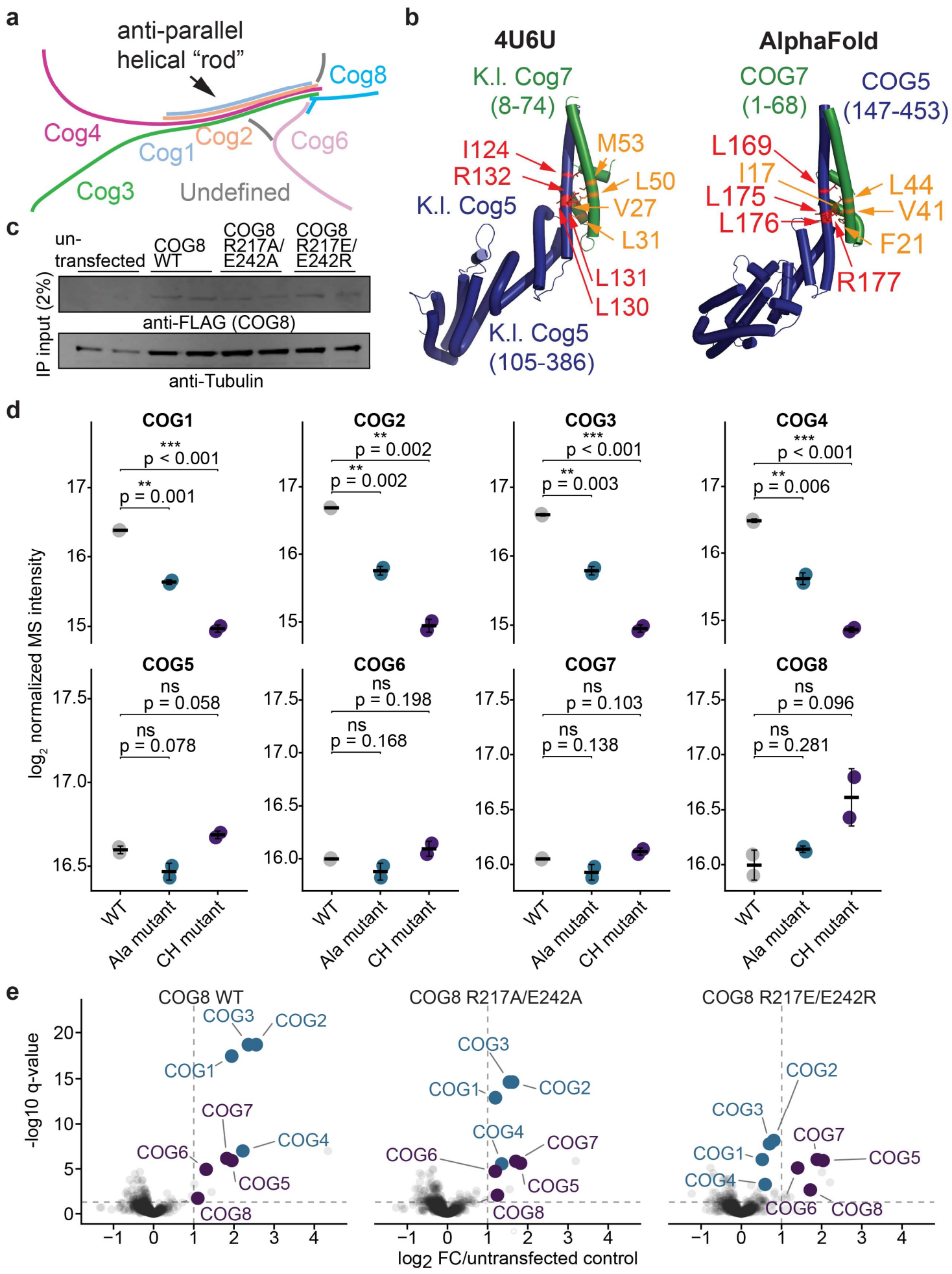

Supplementary Figure 5. Analysis of the COG complex. **a**, Schematic representation of general architecture of the yeast COG complex from negative stain EM experiments[66,67]. **b**, Comparison of Cog5 (residues 105-386)-Cog7 (residues 8-74) crystal structure[68] with the AF-M prediction of the same regions of COG5 (residues 147-453) and COG7 (residues 1-68). Residues previously identified to underly the interaction are indicated[68]. **c**, Relative abundance of COG1-4 (subcomplex I) and COG5-8 (subcomplex II) subunits in duplicate α-Flag immune complexes from HEK293 cells expressing $COG8^{R217E/E242R}$ or $COG8^{R217A/E242A}$ mutants relative to WT COG8-Flag. **d**, TMT signal for WT and mutant COG8 in α-Flag immune complexes. **e**, Volcano plots (log2FC WT or mutant/Control versus q-value) for WT, $COG8^{R217A/E242A}$, and $COG8^{R217E/E242R}$ COG8-Flag immune complexes analyzed by TMT proteomics (in biological duplicate). COG5-8 proteins are indicated in violet and COG1-4 proteins are indicated in blue.

bioRxiv preprint doi: https://doi.org/10.64898/2026.10.29.758816; this version posted October 30, 2026. The copyright holder for this preprint (which was not certified by peer review) is the author/funder, who has granted bioRxiv a license to display the preprint in perpetuity. It is made available under a CC-BY 4.0 International license.

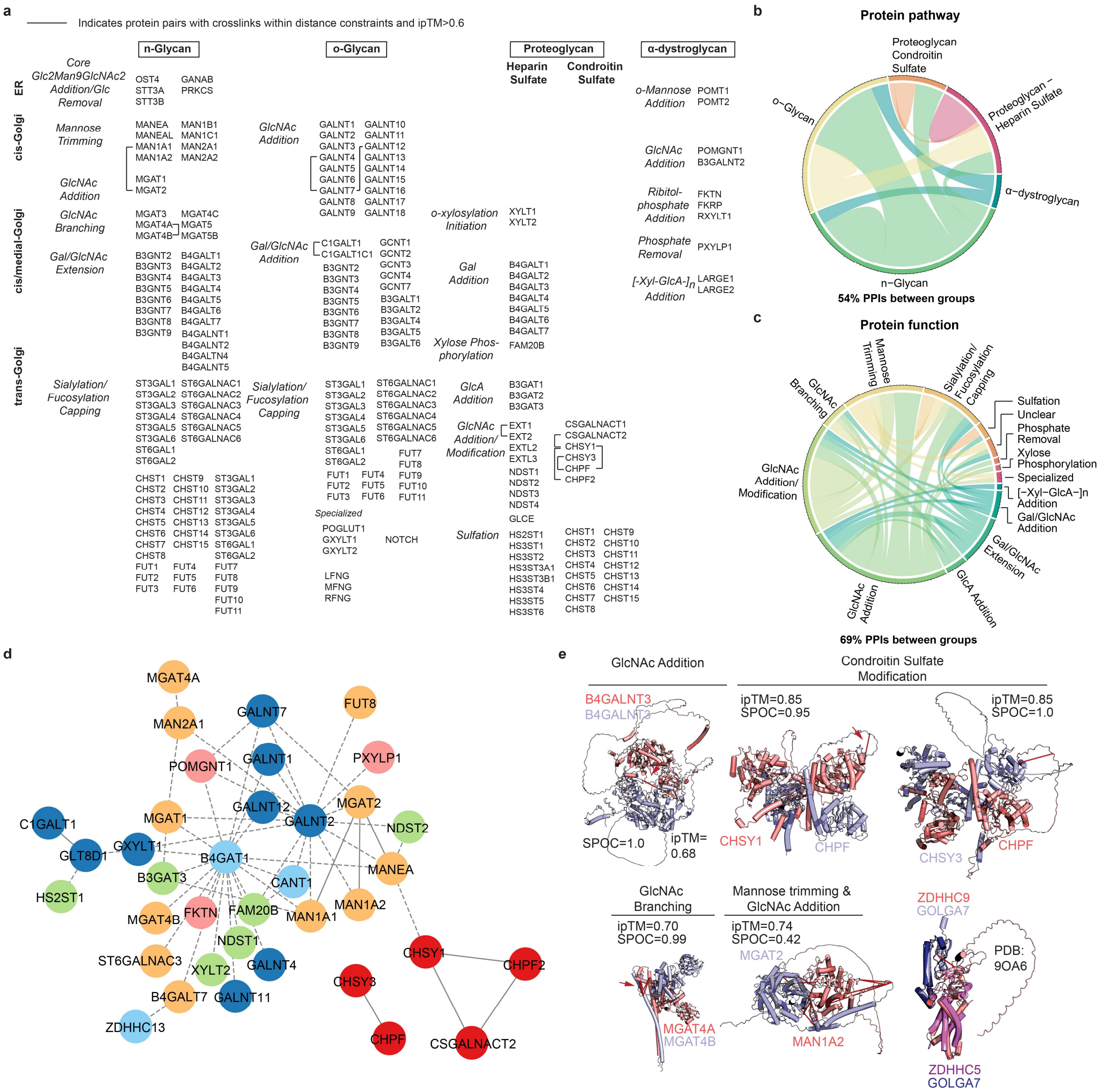

Supplementary Figure 6. Analysis of Golgi matrix enzymes within GolgiFold. a, Summary of n-glycan, o-glycan, proteoglycan, and α-dystroglycan enzymes. Enzymes are organized broadly based on enzymatic function. Lines connecting proteins indicate pairwise combinations with ipTM scores > 0.6 and crosslinks within distance constraints. **b,c**, Annotation of Golgi matrix protein-protein interactions from our Golgi protein compendium organized based on protein pathway (panel **b**) and protein function (panel **c**). **d**, Interaction map of Golgi matrix enzymes identified by XL-MS of purified Golgi. Solid lines indicate ipTM>0.6 and dotted lines indicate ipTM<0.6. **e**, Examples of candidate heterodimers of Golgi matrix enzymes with ipTM scores>0.6 and crosslinks within distance constraints. ipTM (black font) and SPOC (gray font) scores are indicated for each pairwise prediction.
